# Precipitation Mediates the Distribution but Not the Taxonomic Composition of Phytoplankton Communities in a Tributary of Three Gorges Reservoir

**DOI:** 10.3390/plants10091800

**Published:** 2021-08-29

**Authors:** Chengrong Peng, Hongjie Qin, Kan Wang, Yonghong Bi

**Affiliations:** 1 Key Laboratory of Algal Biology, Institute of Hydrobiology, Chinese Academy of Sciences, Wuhan 430072, China; pengcr@ihb.ac.cn; 2 Environmental Horticulture Research Institute, Guangdong Academy of Agricultural Sciences/Guangdong Key Lab of Comprehensive Innovative Utilization of Ornamental Plant Germplasm, Guangzhou 510640, China; hongjieqin111@126.com; 3 Central-Southern Safety & Environment Technology Institute Co., Ltd., Wuhan 430051, China; kan49056@gmail.com

**Keywords:** precipitation, phytoplankton bloom, distribution, mixing regime, spatiotemporal difference

## Abstract

Precipitation is a driver of changes in the spatiotemporal distribution of phytoplankton communities. The ecological consequence of precipitation is important, but the underlying processes are not clear. Here we conducted an immediate prior- and after-event short-interval investigation in the Three Gorges Reservoir region, to test whether the short-term changes in the phytoplankton communities and functional groups could be predicted based on the precipitation level. We found that precipitation of moderate and high levels immediately changed the phytoplankton distribution and altered functional groups. According to structural equation model, the vertical velocity (λ = −0.81), light availability (Zeu/Zmix, λ = 0.47) and relative water column stability (RWCS, λ = 0.38) were important parameters for phytoplankton distribution during the precipitation event. Water quality did not directly affect phytoplankton distribution (λ = −0.11) and effects of precipitation on the water quality only lasted 1–2 days. The phytoplankton community was redistributed with some tolerance functional groups appearance, such as groups F, Lo, M and groups M, MP, TB, W1 appeared during- and after- precipitation event, respectively. We also found that mixing rather than flushing was the driving force for the decrease of phytoplankton biomass. Our study provided valuable data for reservoir regulation and evidence for predictions of phytoplankton during the precipitation events under different climate change scenarios.

## 1. Introduction

Phytoplankton are essential organisms of aquatic food webs, but they can reach unusually high concentrations under suitable conditions. Phytoplankton blooms are becoming increasingly common in aquatic ecosystems worldwide [1]. The dynamics and maximal biomass of phytoplankton are driven by a wide range of factors including abiotic factors such as hydrological conditions and biotic variables like the presence of filter-feeders [2,3]. As a result, the distribution of phytoplankton is site-specific and notoriously patchy and dynamic [4]. Distribution is often disturbed by factors such as precipitation or wind over short-term scales [5,6,7]. Understanding the ecological consequences of phytoplankton community and distribution change in the water column caused by different variables acting on spatial and temporal scales is a challenge for controlling ecosystem productivity [7]. Previous studies have reported that precipitation can change phytoplankton community structure [8,9,10] and succession [11], and can delay the outbreak of phytoplankton blooms [12]. The physical processes by which precipitation changes phytoplankton aggregation in space and in time are not entirely clear, especially in the Three Gorges Reservoir (TGR) region, China, which is one of the largest reservoirs in the world and has experienced frequent phytoplankton blooms since completion of the dam in 2003.

The effects of precipitation on freshwater ecosystems have received increasing attention in recent decades, because extreme precipitation events are predicted to increase due to climate change in the near future [13], and more extreme precipitation events are now being observed globally [14,15]. Freshwater ecosystems in China are directly influenced by the East Asian monsoon, which drives concentrated precipitation spikes in summer and might play a key role in influencing water quality and aquatic biota [16]. Some studies have examined the relationship between precipitation and phytoplankton [9,15,17,18], and precipitation and water quality [19]. The fields observation also displayed a disappearance of phytoplankton bloom and decrease of biomass after precipitation. A question that remains unanswered is how precipitation regulates phytoplankton assemblage and distribution. Additionally, previous studies were limited to rivers, shallow lakes, or small reservoirs as study systems, where precipitation can strongly affect phytoplankton assemblage through flushing and changes in selection pressures such as nutrient concentrations or mixing depth [15,20,21], due to the high fluidity or limited storage capacity of the water body. In large and deep reservoirs, precipitation events may have different impacts on phytoplankton assemblage and dynamics [22,23]. Precipitation is difficult to predict accurately; a rigorous and immediate prior- and after-event short-interval sampling program is required to measure its effects. The potential for global climate change highlights the importance of understanding the ecological consequences of precipitation in terms of the structure and function of aquatic ecosystems in the TGR region and other large water bodies. Huge, deep reservoirs are of particular ecological interest as 57,000+ large dams have been constructed on half of the Earth’s major rivers.

Phytoplankton are sorted into functional groups based on their ecological and physiological traits rather than common morphological characteristics or phylogenetic origins; the functional groups concept better characterizes their role in biogeochemical cycles and reflects environmental changes [24,25], such as *Microcystis* from Group M and *Merismopedia* from Group Lo survive in distinct adaptive strategies with different favor habitat, but they belong to same taxa, Chrococcaceae of Cyanophyta. The phytoplankton structure of the TGR and its relationship to water management and flood regulation has been previously described, with different functional groups dominating during the stratification and mixing seasons [26,27]. However, the effect of precipitation events, including that of the spikes associated with the annual East Asian monsoon, has yet to be adequately measured.

In this study, we describe an in situ timely sampling program in a tributary of TGR during the cyanobacteria bloom period (about 10 days), and test whether short-term changes in the phytoplankton assemblage and functional groups can be predicted from precipitation amount. We tested the hypotheses that: (a) precipitation events would rapidly change the distribution of the phytoplankton assemblage and functional groups; (b) precipitation would result in a loss of phytoplankton biomass; and (c) taxonomic composition of phytoplankton communities differ between prior- and after- precipitation events.

## 2. Materials and Methods

### 2.1. Sampling Site and Sampling Methods

**Figure 1:**
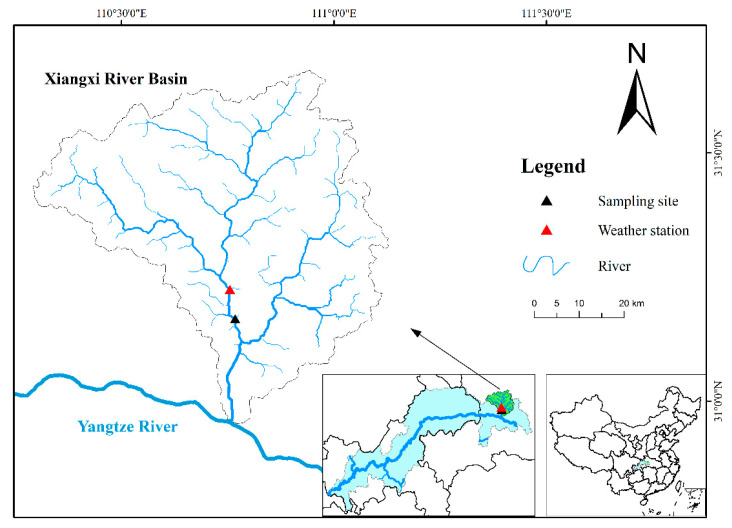
Location of sampling site and weather station in the Xiangxi River of Three Gorges Reservoir.

This study was performed in the Xiangxi River, a tributary of the TGR and eventually discharging into the Yangtze River. It has a watershed of 3095 km^2^, annual average flow 47.4 m^3^/s [28], and annual precipitation ranging from 670 mm to 1700 mm [29]. Daily precipitation and wind data were obtained from the nearest official weather station of Xingshan, which was about 5 km from the sampling site (Figure 1). During a *Microcystis* spp. dominated cyanobacteria bloom (surface biomass of phytoplankton 6.43 ± 3.78 mg L^−1^) in summer, phytoplankton and water quality parameters were measured every day at the sampling site in triplicate (Figure 1), and data of 10 consecutive days were selected to analyze once continuous precipitation appeared.

Water samples were collected from depths of 0.5, 1.0, 2.0, 5.0, and 10.0 m below the water surface, and the water quality parameters of each depth were measured synchronously in situ. Water temperature (WT) and dissolved oxygen (DO) were measured with a YSI Professional Plus (YSI Incorporated, Yellow Springs, OH, USA). The photosynthetically active radiation (PAR) in the air and underwater was measured with a LI-1400 data logger (LI-COR, Lincoln, NE, USA). The flow fields of the sampling sites were surveyed with FlowQuest 600 (LinkQuest Incorporated, San Diego, CA, USA) installed on a boat. Three-dimensional velocity and discharge at the sampling site were analyzed with the FlowQuest 600 Discharge Measurement 6.0.0 package with the offline analysis according to the user’s manual.

Total nitrogen (TN) and permanganate index (COD_Mn_) were determined in accordance with standard methods for water and wastewater [30]. The 1 L bulk water samples for phytoplankton analysis were preserved with 1.5% Lugol solution and concentrated to 30 mL after sedimentation for more than 48 h, then counted in plankton chamber with an optical microscope (Olympus CX21, Tokyo, Japan) under ×400 magnification. Phytoplankton were identified according to algal taxonomy keys [31,32]. The counting and identification were carried out in triplicate with checking of the whole chamber. Mean biovolume (organism mm^3^ L^−1^) of main taxa was calculated by assigning geometric shapes to each cell or filament [33], and assuming the biomass unit as expressed in mass, where 1 mm^3^ L^−1^ = 1 mg L^−1^ [34]. Phytoplankton were classified into functional groups, using the criteria established by Reynolds et al. [24] and Padisák et al. [35].

### 2.2. Data Analysis

In order to assess the immediate effect of precipitation on water quality, a minimum water quality index (*WQI_min_*) method was established according to the equation below [36]:

(1) $$WQI_{min}=\sum_{i=1}^{n}\frac{C_{i}}{n}$$

where *n* is the total number of parameters and *Ci* is the value after normalization. In this study, DO, TN, and COD_Mn_ were normalized based on normalization factors and used to calculate the *WQI_min_* (*n* = 3), following the methods of a water quality assessment at Lake Taihu, China, a large lake at a similar latitude, where *WQI_min_* values were positively correlated with water quality [37].

The euphotic zone (Z_eu_) was calculated as the depth where underwater PAR is 1% of its surface strength [38]. A minimum temperature gradient of 0.2 °C over the depth spacing of the temperature profiles was used to identify the mixing depth (Z_mix_) [39]. The ratio between the euphotic zone and the mixing zone (Z_eu_/Z_mix_) was used as a measure of light availability [40].

The dimensionless parameter of relative water column stability (RWCS) was used to describe the hydrodynamic conditions, and calculated according to the following formula [41]:

(2) $$RWCS=\frac{D_{b}-D_{S}}{D_{4}-D_{5}}$$

where *D_b_* is the density of bottom waters; *D_s_* is the density of the surface waters; and *D*_4_ and *D*_5_ are the densities of pure water at 4°C and 5°C, respectively.

Morisita’s index was used to evaluate the distribution of phytoplankton in the water column. The index was calculated as [42,43]:

(3) $$I_{\delta }=N\cdot \frac{\left(\sum_{i=1}^{N}Xi^{2}-\sum_{i=1}^{N}Xi\right)}{\left[{\left(\sum_{i=1}^{N}Xi\right)}^{2}-\sum_{i=1}^{N}Xi\right]}$$

where *N* is the total number of layers in the water column; *Xi* is the number of individuals in the *i*th layer. The index is equal to 1 for a random distribution, less than 1 for a uniform distribution, and greater than 1 for a clumped distribution.

### 2.3. Statistical Analysis

**Figure 2:**
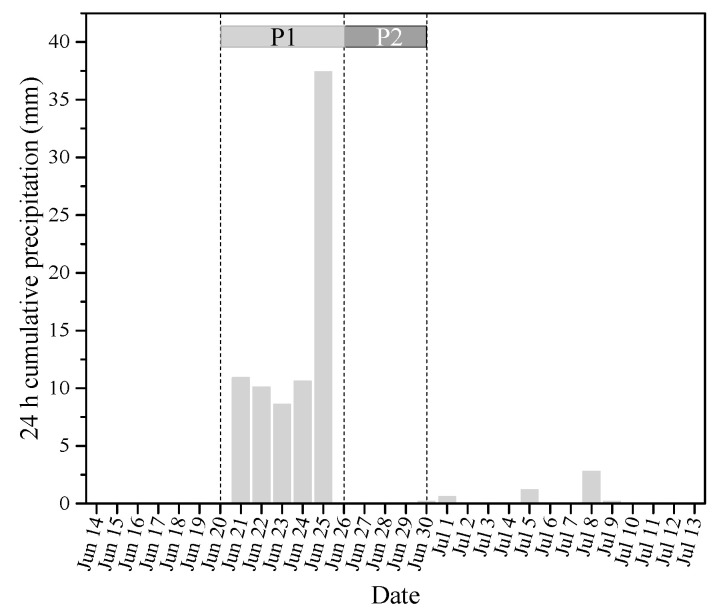
Daily precipitation at the sampling site during the study period.

Based on the precipitation events, the sampling days were divided into two periods (Figure 2): the continuous precipitation period (P1) which included moderate precipitation (Jun 21–Jun 24) and heavy precipitation (Jun 25) days, and the five-day post-precipitation period (P2; Jun 26–30). The precipitation effect is believed to persist for 3–5 days [44]. 

The significant dissimilarities of phytoplankton assemblage structure between P1 and P2 were tested by applying analysis of similarity (ANOSIM) based on permutation procedures with 999 runs [45]. ANOSIM was carried out with the software package Primer 6.0. The differences of selected parameters were separately compared with P1 and P2 using a Wilcoxon rank-sum test. Time-series analysis of a cross-correlation statistical method was used to show time lags of the influence of precipitation on selected parameters [44,46]. Statistical analysis was carried out in the IBM SPSS Statistics 25 package. To characterize the variation of functional groups during- and after- precipitation events, coefficient of variation (CV) was calculated based on standard deviation divided by the mean value. CV values for each functional group were calculated and used to identify variation of functional groups during- and after- precipitation events. High CV means the presence of strong distribution heterogeneity. Low CV indicates low cohort heterogeneity in relation to distribution.

Structural equation model (SEM) analyses were used to analyze the significance of the hypothesized causal relationships among precipitation, water quality (*WQI_min_*), hydrologic regime (velocity, RWCS, Z_eu_/Z_mix_), and phytoplankton assemblage distribution (*I_δ_*). The best-fit model was obtained by using maximum likelihood estimation and improved iteratively by modification in prior models according to a set of modification indices, such as chi-square test (χ^2^), *p* values, degrees of freedom(df), goodness-of-fit index (GFI), and root mean square errors of approximation (RMSEA) [47]. SEM analyses were performed using the IBM Amos 24 package.

## 3. Results

### 3.1. Effects of Precipitation on Water Quality

**Figure 3:**
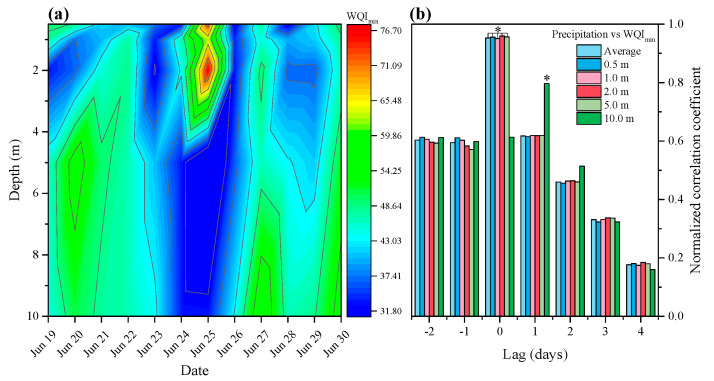
(**a**) Spatiotemporal variation in *WQI_min_* at the sampling site during the study period; (**b**) Time lagged cross-correlation between precipitation and *WQI_min_* at different depths. Asterisks indicate precipitation and corresponding parameter change with a time lag (days).

The *WQI_min_* fluctuated during the observation period, ranging from 31.8 to 76.7 (Figure 3a), representing trophic state indices from hypereutrophic to mesotrophic, and the overall *WQI_min_* showed significant change between P1 and P2 (Wilcoxon tests, *p* < 0.05). Before the five-day precipitation event, the sampling site was experiencing a cyanobacteria bloom (surface biomass of phytoplankton 5.52 ± 2.98 mg L^−1^), which was dominated by *Microcystis*, and the spatial distribution of *WQI_min_* was uneven across different depths, with a relatively low average *WQI_min_* of 45.1. The *WQI_min_* decreased, with trophic state worsening, with the continuous moderate precipitation. The highest peak was observed 2 m below the water surface during heavy precipitation (Jun 25). After precipitation, the average *WQI_min_* was much higher, though the spatial distribution of *WQI_min_* was uneven in the water column. Cross-correlation indicated that the water quality of the upper layer (0–5 m) increased the day of precipitation, but that of the lower layer (5–10 m) increased 1 day after the precipitation event (Figure 3b). The effects of precipitation on the water quality lasted 1–2 days, then the water column gradually reverted to the pre-precipitation state.

### 3.2. Effects of Precipitation on Hydrodynamics

**Figure 4:**
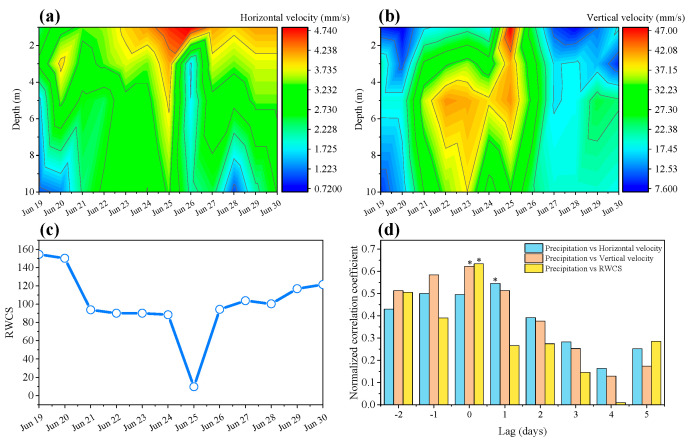
Spatiotemporal variation in (**a**) horizontal velocity, (**b**) vertical velocity, and (**c**) relative water column stability at the sampling site during the study period; (**d**) time lagged cross-correlation between precipitation and horizontal velocity, vertical velocity, and RWCS. Asterisks indicate precipitation and corresponding parameter change with a time lag (days).

During this study, the maximum wind speed reached 12.3 m s^−1^, but the mean wind speed was only 1.1 m s^−1^, with the main wind direction from the south-southwest (Figure S1). The horizontal and vertical velocity in the water column showed different patterns, with the vertical velocity being much higher than horizontal velocity during the study period (Figure 4a,b). The horizontal velocity at different depths in the water column remained relatively stable during rainy days, even during heavy precipitation, and the overall horizontal velocity showed no significant change between P1 and P2 (Wilcoxon tests, *p* > 0.05). However, the vertical velocity at different depths in the water column varied greatly during rainy days, especially in the upper layer, in which it increased almost two times during heavy precipitation, and vertical velocity changed significantly between P1 and P2 (Wilcoxon tests, *p* < 0.05). The RWCS decreased as the water column started mixing across the precipitation period (Figure 4c) and became almost completely mixed during the heavy precipitation day. Stratification resumed 1 day after the precipitation disturbance and RWCS showed significant change between P1 and P2 (Wilcoxon tests, *p* < 0.05). Cross-correlation indicated that precipitation affected flow field and stratification of the water column at different times. The vertical velocity increased and RWCS decreased the day of precipitation, while the horizontal velocity changed 1 day after the precipitation event (Figure 4d). This freshwater probably reached the sampling site 1 day after precipitation (Figure 4d). The RWCS decreased as the mixing increased: the water column started mixing the day of precipitation (Figure 4d), and almost completely mixed in the heavy precipitation day (Figure 4c).

### 3.3. Phytoplankton Assemblage Dynamics

**Figure 5:**
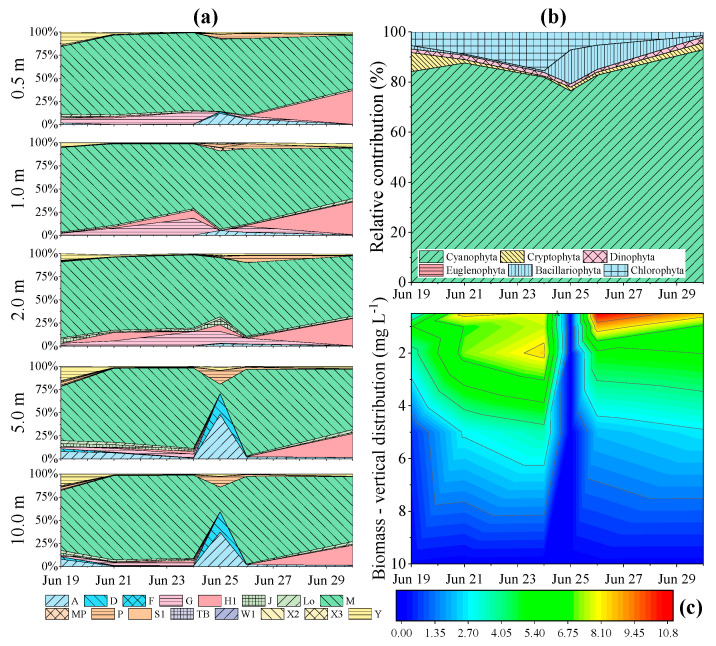
Variation of phytoplankton assemblage in the Xiangxi River during the study period. (**a**) Vertical distribution of phytoplankton function groups; (**b**) Relative contribution of phytoplankton; (**c**) Vertical distribution of phytoplankton biomass.

During the study period, the phytoplankton assemblage was dominated by *Microcystis* spp., and a total of 36 algal taxa belonging to 6 phyla were recorded. Sixteen functional groups were classified, including the 28 descriptor taxa (Table S1). The M, H1, G, A, and Y functional groups were the main contributors to the phytoplankton assemblage in the Xiangxi River across the study period (Figure 5a). Before the precipitation event, the phytoplankton community was dominated by M and H1 functional groups, but there was marked temporal and spatial variation in the representation of the functional groups of phytoplankton during rainy days (Figure 5a). Group Y sharply decreased in the water column after the start of precipitation. During the heavy precipitation day, the phytoplankton community was dominated by Groups M, A, and G, and the deeper layer of the water column was dominated by Groups A, D, P, and M. The overall phytoplankton assemblage structure showed no detectable change between P1 and P2 (ANOSIM, *p* > 0.05). The dominant taxon (with the highest cell density) was cyanobacteria over the entire course of the study, with the proportion of cyanobacteria remaining higher than that of the other taxa (Figure 5b). After the precipitation event, the proportion of bacillariophyta increased slowly, but this phenomenon just last 3 days. The vertical distribution of phytoplankton biomass changed significantly during the precipitation period (Figure 5c). The biomass was higher in the upper layer than in the deeper layer during the continuous moderate precipitation period, while it became very low in the entire water column during the heavy precipitation day. However, the distribution of phytoplankton recovered quickly from this stage after the cessation of heavy precipitation, with the biomass increasing, and even being higher, in the upper layer than before precipitation occurred.

For our study, in most functional groups the values of CV are rather high (Table S2), indicating heterogeneity of the spatiotemporal distribution. Groups F, Lo, M and groups M, MP, TB, W1 shared the last 20% average CV values at all sampling depths in the P1 and P2, respectively (Table S2), indicated their relative stability along the time course. Group M (i.e., cyanobacteria) could persist during and after precipitation events, even after ca. 80 mm precipitation in 5 days.

**Figure 6:**
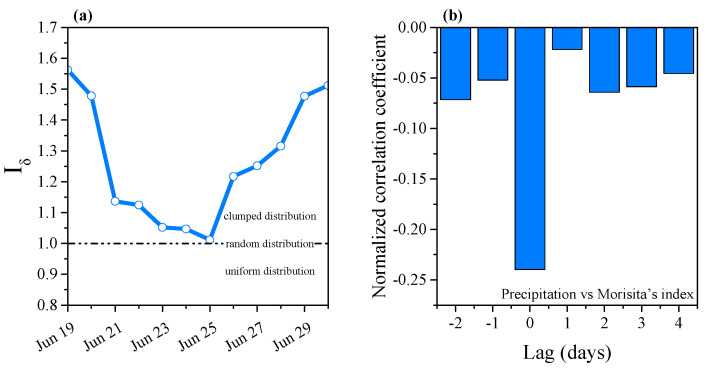
(**a**) Temporal variation in Morisita’s index during the study period; (**b**) Time-lagged cross-correlation between precipitation and Morisita’s index.

Distribution of phytoplankton in the water column was affected by the precipitation event. Before the precipitation period, Morisita’s index was higher than during the precipitation period, indicating that the phytoplankton had a clumped distribution (Figure 5c and Figure 6). During the continuous precipitation, Morisita’s index decreased over time. The lowest value was observed during heavy precipitation; the value was close to 1, revealing that the vertical distribution of phytoplankton was significantly affected by the precipitation. Phytoplankton was randomly distributed during this time. After the rainy period, the distribution of phytoplankton returned to a clumped distribution. Corresponding to the cross-correlation coefficient, the lag was negative, indicating no direct significant effect of precipitation on Morisita’s index (Figure 6b).

### 3.4. Structural Equation Model (SEM)

**Figure 7:**
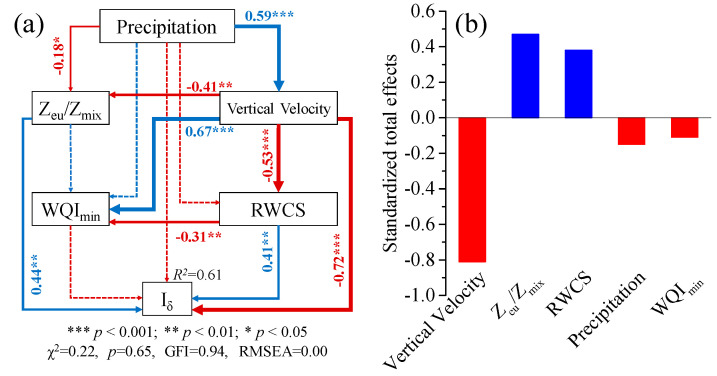
The direct (**a**) and total (**b**) effects of five variables on phytoplankton distribution, as determined by structural equation modeling. Arrow width and the numbers on the arrows correspond to the standardized path coefficients; significant and nonsignificant path coefficients are indicated by solid and dotted lines, respectively; blue and red arrows indicate positive and negative flows of causality (*p* < 0.05), respectively.

The fitting parameters of all minimal adequate path analysis explained 61% of the variance in phytoplankton distribution (Figure 7a). Vertical velocity (λ = −0.81) was the strongest predictor of phytoplankton distribution (Figure 7b) and was positively driven by precipitation (*r* = 0.59, *p* < 0.001). The vertical velocity directly affected phytoplankton distribution (*r* = −0.72, *p* < 0.001), also strongly explained the variance of RWCS and Z_eu_/Z_mix_, which directly contributed to the phytoplankton distribution in the water column (Figure 7a).

## 4. Discussion

Precipitation governs water quality variation in river systems, especially when the river is regulated by dams [19,48]. But there is a knowledge gap in the physical processes by which precipitation changes phytoplankton aggregation in space and in time. Surface water nutrient concentrations often increase markedly during and immediately after precipitation events [49,50]. Nutrients from precipitation-runoff lead to the deterioration of water quality in the TGR basin. This phenomenon was observed in the current study, where continuous moderate precipitation increased the concentration of many nutrients in the water column (unpublished data), and the *WQI_min_* decreased (Figure 3a). However, the water quality of surface water increased during heavy precipitation, which may be due to a dilution effect. Although *WQI_min_* increased slightly during heavy precipitation, it returned to pre-precipitation values quickly, and even continued to decrease. Water quality variation was observed 0 and 1 day following precipitation at the depths of 0–5 m and 10 m, respectively (Figure 3b). The results of cross-correlation statistical analysis imply that water quality synchronized with discharge after precipitation, which is the main cue for dynamics of phytoplankton population during the summer season [44]. The possibility that the East Asian monsoon summer rains drive phytoplankton dynamics in the TGR deserves further study.

Wind plays an important role in the distribution of phytoplankton by mixing the surface layer [4,28,51]. The strength and effect of these shear forces depend on the wind speed [4,52,53]. The patchiness of phytoplankton in lakes and reservoirs disappears at wind speeds above 3–4 m s^−1^ [5,54]. The influence of winds on mixing of the surface layer is small: the horizontal velocity at all depths in the water column remained relatively low during rainy days, even during heavy precipitation (Figure 4a,b). Mixing regime governs the phytoplankton composition [55]; the structure of phytoplankton communities is mainly determined by resource availability [56] and hydrological conditions [4,51]. Hydrological conditions are integral drivers of community assemblages in short-term weather events. During the precipitation days, mixing may have been selected for groups tolerant to mixing regime and low light, such as groups F, Lo, and M. After the precipitation event, groups M, MP, TB, W1 were more stable than other groups. Contrarily to the traditional paradigm that short-term and abrupt changes in water column attenuate cyanobacterial blooms, the results showed that in some cases (after ca. 80 mm precipitation in 5 days), cyanobacteria (i.e., group M) can persist over time.

During the study period, the dominant taxon in the Xiangxi River was cyanobacteria, primarily *Microcystis* spp., and accounted for nearly 90% of the cell density. Previous research showed that an intense East Asian monsoon reduced cyanobacterial bloom while a weak monsoon increased it [57]. However, our data suggested that phytoplankton concentrated in the upper layer of the water column after continuous moderate precipitation, resulting in low cell density of phytoplankton in the deeper layer of the water column. Cell density of phytoplankton in the entire water column was significantly lower on the day of heavy precipitation than during other days (Figure 5c), providing partial support for our second hypothesis. However, cell density recovered and proliferated from the precipitation event quickly, faster than other studies have reported [58,59]. There are several potential explanations for this rapidity: an increased concentration of nutrients in the upper layers of the water column after moderate rain drew phytoplankton there (evidenced by their clumped distribution), and/or subsequent heavy rains caused phytoplankton to migrate horizontally and vertically due to the destabilization of the water column (resulting in random distribution; Figure 6).

The most important precondition for a cyanobacteria bloom is the water column stability [60]. Previous studies showed that the phytoplankton diversity was low during blooms [9,61]. Our study similarly found that during the cyanobacteria bloom, phytoplankton diversity in the water column was also low. When the hydrological conditions of Xiangxi River were significantly affected by heavy precipitation, the cyanobacteria bloom disappeared (Figure 5c). We observed changes in phytoplankton distribution after the precipitation event, in support of our first hypothesis. The physical disturbance caused by heavy precipitation may generate a uniform phytoplankton distribution in the water column and enable benthic taxa to co-exist in surface water [9]. This positively affects phytoplankton diversity, and a tendency of diversity to increase at lower biomass was also observed [62]. Similarly, during the heavy precipitation period of this study, phytoplankton diversity slightly increased in the lower water column even though the biomass decreased. During moderate continuous precipitation, Morisita’s index decreased, suggesting that the distribution of phytoplankton in the water column was affected (Figure 6); however, there were no obvious changes in phytoplankton composition structure during the continuous precipitation period (Figure 5a).

Precipitation can abruptly affect environmental conditions and community assemblages. In the current study, a precipitation event altered water quality and phytoplankton distribution. However, no overarching changes in the phytoplankton taxonomic composition were found during the study period. This is likely because cyanobacteria overwhelmingly dominated the community (>90%), while other taxa were scarce. The reason for the disappearance of the cyanobacteria bloom during heavy precipitation is the phytoplankton vertical migration driven by vertical velocity (Figure 7). The measured water quality parameters and phytoplankton biomass returned to pre-rain levels quickly (Figure 3a and Figure 5c), after sedimentation of suspended particles and an increase in light availability. Precipitation has the potential to show long-term effects on aquatic ecosystems, in particular there may be a massive phytoplankton bloom after precipitation events due to the high input of nutrients and light availability.

After the completion of the dam at the TGR, ecological changes in tributary backwaters have attracted widespread attention and study due to the high incidence of phytoplankton blooms [63]. Some studies have indicated that a mixing regime caused by sufficiently large water level fluctuations might be an effective way to inhibit phytoplankton blooms [28,64,65]. Unfortunately, it is impossible for the TGR to maintain regular, sufficiently large water level fluctuations and meet the goals of flood management, water resource supplies, and hydropower production. Flood-operations and flow-operations are carried out each year in the TGR according to the Directive of the Ministry of Water Resources of the People’s Republic of China; however, these hydrological approaches are based on flow, without consideration of biology. A practical approach for phytoplankton bloom or productivity control is still needed. Our results suggest that under future climate change scenarios, precipitation might be a valuable signal for reservoir regulation due to the widespread climate monitoring network that makes it easy to obtain real-time precipitation information, and timely flow-operation can flush more phytoplankton when they are mixed by precipitation, limiting harmful blooms.

## Data Availability

The data presented in this study are available upon request from the corresponding author.

## Supplementary Materials

The following are available online at https://www.mdpi.com/article/10.3390/plants10091800/s1, Figure S1: Wind direction and speed in the Xiangxi River watershed during the study period. Table S1: Main phytoplankton functional groups and representative taxa recorded during the study period from Xiangxi River. ** The dominant taxon. Table S2: The CV value of each functional group at each sampling depth. Bold indicates the last 20% of the CV value at all sampling depth. NA means not available, for the corresponding functional group were not detected in that period.

## References

[1] Chen N.W., Mo Q.L., Kuo Y.M., Su Y.P., Zhong Y.P. (2018). Hydrochemical controls on reservoir nutrient and phytoplankton dynamics under storms. Sci. Total Environ..

[2] Havens K.E., Ji G., Beaver J.R., Fulton R.S., Teacher C.E. (2017). Dynamics of cyanobacteria blooms are linked to the hydrology of shallow Florida lakes and provide insight into possible impacts of climate change. Hydrobiologia.

[3] Kuo Y.M., Wu J.T. (2016). Phytoplankton dynamics of a subtropical reservoir controlled by the complex interplay among hydrological, abiotic, and biotic variables. Environ. Monit. Assess..

[4] Cyr H. (2017). Winds and the distribution of nearshore phytoplankton in a stratified lake. Water Res..

[5] Vidal J., Rigosi A., Hoyer A., Escot C., Rueda F.J. (2014). Spatial distribution of phytoplankton cells in small elongated lakes subject to weak diurnal wind forcing. Aquat. Sci..

[6] Yang J.R., Lv H., Isabwe A., Liu L.M., Yu X.Q., Chen H.H., Yang J. (2017). Disturbance-induced phytoplankton regime shifts and recovery of cyanobacteria dominance in two subtropical reservoirs. Water Res..

[7] Serra T., Vidal J., Casamitjana X., Soler M., Colomer J. (2007). The role of surface vertical mixing in phytoplankton distribution in a stratified reservoir. Limnol. Oceanogr..

[8] Jeong K.S., Kim D.K., Joo G.J. (2007). Delayed influence of dam storage and discharge on the determination of seasonal proliferations of *Microcystis aeruginosa* and *Stephanodiscus hantzschii* in a regulated river system of the lower Nakdong River (South Korea). Water Res..

[9] Hong S.-S., Bang S.W., Kim Y.O., Han M.S. (2002). Effects of rainfall on the hydrological conditions and phytoplankton community structure in the riverine zone of the Pal’tang Reservoir, Korea. J. Freshwater Ecol..

[10] Ahn C.Y., Chung A.S., Oh H.M. (2002). Rainfall, phycocyanin, and N: P ratios related to cyanobacterial blooms in a Korean large reservoir. Hydrobiologia.

[11] Znachor P., Zapomelova E., Rehakova K., Nedoma J., Simek K. (2008). The effect of extreme rainfall on summer succession and vertical distribution of phytoplankton in a lacustrine part of a eutrophic reservoir. Aquat. Sci..

[12] Iriarte A., Purdie D.A. (2004). Factors controlling the timing of major spring bloom events in an UK south coast estuary. Estuar. Coast. Shelf S..

[13] IPCC (2013). Climate Change 2013: The Physical Science Basis.

[14] Lehmann J., Coumou D., Frieler K. (2015). Increased record-breaking precipitation events under global warming. Clim. Chang..

[15] Richardson J., Feuchtmayr H., Miller C., Hunter P.D., Maberly S.C., Carvalho L. (2019). Response of cyanobacteria and phytoplankton abundance to warming, extreme rainfall events and nutrient enrichment. Glob. Chang. Biol..

[16] Guo C.X., Zhu G.W., Paerl H.W., Zhu M.Y., Yu L., Zhang Y.B., Liu M.L., Zhang Y.L., Qin B.Q. (2018). Extreme weather event may induce Microcystis blooms in the Qiantang River, Southeast China. Environ. Sci. Pollut. R..

[17] Zhou G., Zhao X., Bi Y., Hu Z. (2012). Effects of rainfall on spring phytoplankton community structure in Xiangxi Bay of the Three-Gorges Reservoir, China. Fresen. Environ. Bull..

[18] Wu T.F., Qin B.Q., Zhu G.W., Luo L.C., Ding Y.Q., Bian G.Y. (2013). Dynamics of cyanobacterial bloom formation during short-term hydrodynamic fluctuation in a large shallow, eutrophic, and wind-exposed Lake Taihu, China. Environ. Sci. Pollut. R..

[19] Jeong K.-S., Kim D.-K., Shin H.-S., Yoon J.-D., Kim H.-W., Joo G.-J. (2011). Impact of summer rainfall on the seasonal water quality variation (chlorophyll a) in the regulated Nakdong River. KSCE J. Civ. Eng..

[20] Sadro S., Melack J.M. (2012). The Effect of an Extreme Rain Event on the Biogeochemistry and Ecosystem Metabolism of an Oligotrophic High-Elevation Lake. Arct. Antarct. Alp. Res..

[21] Badylak S., Phlips E., Dix N., Hart J., Srifa A., Haunert D., He Z.L., Lockwood J., Stofella P., Sun D.T. (2016). Phytoplankton dynamics in a subtropical tidal creek: Influences of rainfall and water residence time on composition and biomass. Mar. Freshwater Res..

[22] Paerl H.W., Gardner W.S., Havens K.E., Joyner A.R., McCarthy M.J., Newell S.E., Qin B.Q., Scott J.T. (2016). Mitigating cyanobacterial harmful algal blooms in aquatic ecosystems impacted by climate change and anthropogenic nutrients. Harmful Algae.

[23] Perga M.E., Bruel R., Rodriguez L., Guenand Y., Bouffard D. (2018). Storm impacts on alpine lakes: Antecedent weather conditions matter more than the event intensity. Glob. Chang. Biol..

[24] Reynolds C.S., Huszar V., Kruk C., Naselli-Flores L., Melo S. (2002). Towards a functional classification of the freshwater phytoplankton. J. Plankton Res..

[25] Yang J., Lv H., Yang J., Liu L.M., Yu X.Q., Chen H.H. (2016). Decline in water level boosts cyanobacteria dominance in subtropical reservoirs. Sci. Total Environ..

[26] Peng C., Zhang L., Zheng Y., Li D. (2013). Seasonal succession of phytoplankton in response to the variation of environmental factors in the Gaolan River, Three Gorges Reservoir, China. Chin. J. Oceanol. Limnol..

[27] Zhu K.X., Bi Y.H., Hu Z.Y. (2013). Responses of phytoplankton functional groups to the hydrologic regime in the Daning River, a tributary of Three Gorges Reservoir, China. Sci. Total Environ..

[28] Liu L., Liu D.F., Johnson D.M., Yi Z.Q., Huang Y.L. (2012). Effects of vertical mixing on phytoplankton blooms in Xiangxi Bay of Three Gorges Reservoir: Implications for management. Water Res..

[29] Han J.C., Huang G.H., Zhang H., Li Z., Li Y.P. (2014). Heterogeneous Precipitation and Streamflow Trends in the Xiangxi River Watershed, 1961-2010. J. Hydrol. Eng..

[30] APHA (2012). Standard Methods for the Examination of Water and Wastewater.

[31] Hu H., Wei Y. (2006). The Freshwater Algae of China: Systematics, Taxonomy and Ecology.

[32] John D.M., Whitton B.A., Brook A.J. (2002). The freshwater algal flora of the British Isles: An identification guide to freshwater and terrestrial algae.

[33] Brierley B., Carvalho L., Davies S., Krokowski J. (2007). Guidance on the Quantitative Analysis of Phytoplankton in Freshwater Samples.

[34] Napiórkowska-Krzebietke A., Kobos J. (2016). Assessment of the cell biovolume of phytoplankton widespread in coastal and inland water bodies. Water Res..

[35] Padisak J., Crossetti L.O., Naselli-Flores L. (2009). Use and misuse in the application of the phytoplankton functional classification: A critical review with updates. Hydrobiologia.

[36] Pesce S.F., Wunderlin D.A. (2000). Use of water quality indices to verify the impact of Cordoba City (Argentina) on Suquia River. Water Res..

[37] Wang J.L., Fu Z.S., Qiao H.X., Liu F.X. (2019). Assessment of eutrophication and water quality in the estuarine area of Lake Wuli, Lake Taihu, China. Sci. Total Environ..

[38] Kirk J.T.O. (1994). Light and Photosynthesis in Aquatic Ecosystems.

[39] Amaral J.H.F., Borges A.V., Melack J.M., Sarmento H., Barbosa P.M., Kasper D., de Melo M.L., De Fex-Wolf D., da Silva J.S., Forsberg B.R. (2018). Influence of plankton metabolism and mixing depth on CO_2_ dynamics in an Amazon floodplain lake. Sci. Total Environ..

[40] Jensen J.P., Jeppesen E., Olrik K., Kristensen P. (1994). Impact of Nutrients and Physical Factors on the Shift from Cyanobacterial To Chlorophyte Dominance in Shallow Danish Lakes. Can. J. Fish Aquat. Sci..

[41] Padisák J., Barbosa F., Koschel R., Krienitz L. (2003). Deep layer cyanoprokaryota maxima in temperate and tropical lakes. Arch. Hydrobiol. Spec. Issues Adv. Limnol..

[42] Thackeray S.J., George D.G., Jones R.I., Winfield I.J. (2006). Statistical quantification of the effect of thermal stratification on patterns of dispersion in a freshwater zooplankton community. Aquat. Ecol..

[43] Hills J.M., Thomason J.C. (1996). A multi-scale analysis of settlement density and pattern dynamics of the barnacle Semibalanus balanoides. Mar. Ecol. Prog. Ser..

[44] Baek S.H., Shimode S., Kim H.C., Han M.S., Kikuchi T. (2009). Strong bottom-up effects on phytoplankton community caused by a rainfall during spring and summer in Sagami Bay, Japan. J. Marine Syst..

[45] Clarke K.R. (1993). Nonparametric multivariate analyses of changes in community structure. Aust. J. Ecol..

[46] Zhang M., Niu Z.P., Cai Q.H., Xu Y.Y., Qu X.D. (2019). Effect of Water Column Stability on Surface Chlorophyll and Time Lags under Different Nutrient Backgrounds in a Deep Reservoir. Water.

[47] Wang D.D., Zhu Z.K., Shahbaz M., Chen L., Liu S.L., Inubushi K., Wu J.S., Ge T.D. (2019). Split N and P addition decreases straw mineralization and the priming effect of a paddy soil: A 100-day incubation experiment. Biol. Fert. Soils.

[48] Wolf K.A., Gupta S.C., Rosen C.J. (2020). Precipitation Drives Nitrogen Load Variability in Three Iowa Rivers. J. Hydrol. Reg. Stud..

[49] Sherson L.R., Van Horn D.J., Gomez-Velez J.D., Crossey L.J., Dahm C.N. (2015). Nutrient dynamics in an alpine headwater stream: Use of continuous water quality sensors to examine responses to wildfire and precipitation events. Hydrol. Process.

[50] Walker J.C.G. (1991). Biogeochemistry - an Analysis of Global Change. Science.

[51] Monismith S.G., MacIntyre S., Likens G.E. (2009). The Surface Mixed Layer in Lakes and Reservoirs. Encyclopedia of Inland Waters.

[52] Boegman L., Likens G.E. (2009). Currents in Stratified Water Bodies 2: Internal Waves. Encyclopedia of Inland Waters.

[53] Kim T.W., Najjar R.G., Lee K. (2014). Influence of precipitation events on phytoplankton biomass in coastal waters of the eastern United States. Glob. Biogeochem. Cycles.

[54] Hunter P.D., Tyler A.N., Willby N.J., Gilvear D.J. (2008). The spatial dynamics of vertical migration by Microcystis aeruginosa in a eutrophic shallow lake: A case study using high spatial resolution time-series airborne remote sensing. Limnol. Oceanogr..

[55] Becker V., Caputo L., Ordonez J., Marce R., Armengol J., Crossetti L.O., Huszar V.L.M. (2010). Driving factors of the phytoplankton functional groups in a deep Mediterranean reservoir. Water Res..

[56] Reynolds C.S. (2006). The Ecology of Phytoplankton.

[57] An K.-G., Jones J.R. (2000). Factors regulating bluegreen dominance in a reservoir directly influenced by the Asian monsoon. Hydrobiologia.

[58] Ye L., Han X., Xu Y., Cai Q. (2007). Spatial analysis for spring bloom and nutrient limitation in Xiangxi bay of three Gorges Reservoir. Environ. Monit. Assess..

[59] Zhou G., Zhao X., Bi Y., Liang Y., Hu J., Yang M., Mei Y., Zhu K., Zhang L., Hu Z. (2011). Phytoplankton variation and its relationship with the environment in Xiangxi Bay in spring after damming of the Three-Gorges, China. Environ. Monit. Assess..

[60] Park H., Jheong W., Kwon O., Ryu J. (2000). Seasonal succession of toxic cyanobacteria and microcystins concentration in Paldang reservoir. Algae.

[61] Jacobsen B.A., Simonsen P. (1993). Disturbance events affecting phytoplankton biomass, composition and species diversity in a shallow, eutrophic, temperate lake. Intermediate Disturbance Hypothesis in Phytoplankton Ecology.

[62] Moustaka-Gouni M. Phytoplankton succession and diversity in a warm monomictic, relatively shallow lake: Lake Volvi, Macedonia, Greece. Intermediate Disturbance Hypothesis in Phytoplankton Ecology.

[63] Ministry of Environmental Protection of China (2019). Bulletin on the Ecological and Environmental Monitoring Results of the Three Gorges Project(2003-2018).

[64] Paillisson J.M., Marion L. (2011). Water level fluctuations for managing excessive plant biomass in shallow lakes. Ecol. Eng..

[65] Yang Z.J., Liu D.F., Ji D.B., Xiao S.B. (2010). Influence of the impounding process of the Three Gorges Reservoir up to water level 172.5 m on water eutrophication in the Xiangxi Bay. Sci. China Technol. Sci..

