# Supplementary material for: Precipitation Mediates the Distribution but Not the Taxonomic Composition of Phytoplankton Communities in a Tributary of Three Gorges Reservoir
Source: Plants (Basel). 2021 Aug 29;10(9):1800. doi: 10.3390/plants10091800 (PMC8466488; doi:10.3390/plants10091800)
Supplement: Supplementary file 1 [file plants-10-01800-s001.zip › plants-1341404-supplementary.pdf]

Supplementary Materials

Precipitation Mediates the Distribution but not the Taxonomic Composition of Phytoplankton Communities in a Tributary of Three Gorges Reservoir

Chengrong Peng [1], Hongjie Qin [2], Kan Wang [3] and Yonghong Bi [1,*]

[1] Key Laboratory of Algal Biology, Institute of Hydrobiology, Chinese Academy of Sciences, Wuhan 430072, China; pengcr@ihb.ac.cn
[2] Environmental Horticulture Research Institute, Guangdong Academy of Agricultural Sciences / Guangdong Key Lab of Comprehensive Innovative Utilization of Ornamental Plant Germplasm, Guangzhou, Guangdong 510640, China; hongjieqin111@126.com
[3] Central-Southern Safety & Environment Technology Institute Co., Ltd., Wuhan 430051, China; kan49056@gmail.com
* Correspondence: biyh@ihb.ac.cn

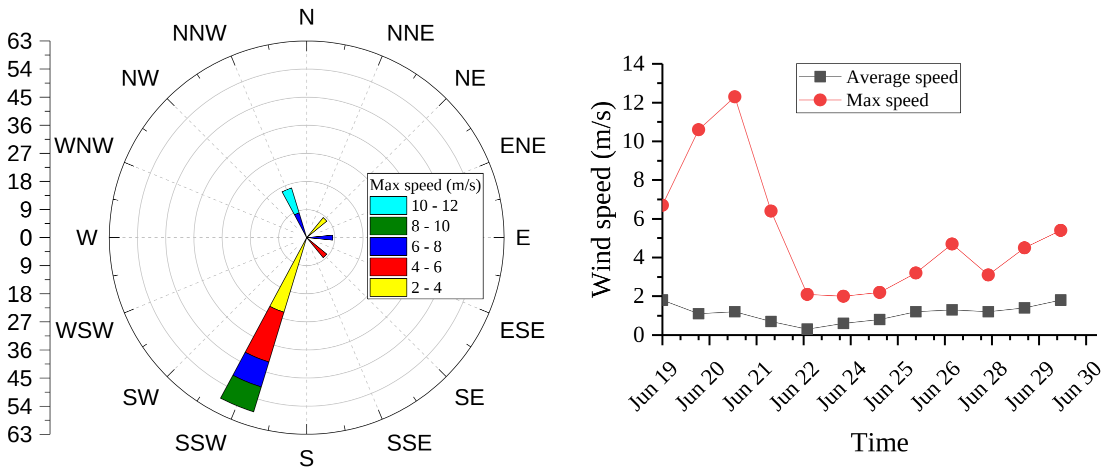

Figure S1. Wind direction and speed in the Xiangxi River watershed during the study period.

Table S1. Main phytoplankton functional groups and representative taxa recorded during the study period from Xiangxi River. ** The dominant taxon.

**Functional group**	**Main phytoplankton species in this study**	**Habitat template** [1,2]
A	*Cyclotella* spp.	Clear, often well-mixed, base poor, lakes
D	*Synedra* spp.	Shallow, enriched turbid waters, including rivers
F	*Ankistrodesmus* spp., *Oocystis* spp.	Clear, deeply mixed meso-eutrophic lakes
G	*Volvox* spp., *Pandorina* spp., *Eudorina* spp.	Stagnating water columns in small eutrophic lakes, reservoirs and stable
H1	*Anabaena* spp.	Dinitrogen-fixing Nostocaleans
J	*Scenedesmus* spp., *Pediastrum* spp., *Coelastrum* spp., *Crucigenia* spp.	Shallow, enriched lakes, ponds, and rivers
L_O	*Ceratoneis* spp., *Peridiniopsis niei, Ceratium hirundinella*	Deep or shallow, oliotrophic or eutrophic, medium to large lakes
M	*Microcystis* spp. **	Dielly mixed layers of small eutrophic, low latitude lakes
MP	*Cocconeis* spp., *Gomphonema* spp., *Cymbella* spp., *Chlorococcales* spp.	Frequently stirred up, inorganically turbid shallow lakes
P	*Melosira* spp., *Navicula* spp.	Eutrophic epilimnia
S1	*Pseudanabeana* spp.	Turbid mixed environments (shade-adapted cyanoprokaryotes)
T_B	*Achnanthes* spp.	Highly lotic environments (streams and rivulets)
W1	*Euglena* spp.	Ponds, even temporary, rich in organic matter from husbandry or sewages
X2	*Chlamydomonas* spp.	Shallow, clear mixed layers in mesoeutrophic lakes
X3	*Schroederia* spp.	Shallow, clear, mixed layers
Y	*Cryptomonas* spp.	Usually, small, enriched lakes

Table S2. The CV value of each functional group at each sampling depth. Bold indicates the last 20% of the CV value at all sampling depth. NA means not available, for the corresponding functional group were not detected in that period.

**Functional Group**	Average		**Depth**									
			**0.5 m**		**1 m**		**2 m**		**5 m**		**10 m**	
	P1	P2	P1	P2	P1	P2	P1	P2	P1	P2	P1	P2
**A**	151.5%	84.0%	164.3%	137.3%	164.0%	105.4%	123.6%	107.8%	139.7%	36.7%	165.6%	32.9%
**D**	170.4%	55.7%	167.0%	141.4%	173.2%	55.3%	173.2%	9.8%	169.1%	31.3%	169.7%	40.8%
**F**	**37.3%**	56.6%	NA	141.4%	NA	NA	NA	141.4%	89.0%	NA	97.5%	NA
**G**	84.9%	82.4%	97.9%	129.3%	106.5%	141.4%	46.6%	141.4%	86.6%	NA	86.7%	NA
**H1**	127.1%	139.9%	173.2%	141.4%	136.2%	134.0%	88.6%	141.4%	121.4%	141.4%	116.0%	141.4%
**J**	69.2%	116.7%	NA	141.4%	NA	122.6%	91.6%	36.4%	89.9%	141.4%	164.7%	141.4%
**Lo**	**59.2%**	61.5%	49.6%	40.3%	5.1%	56.6%	26.2%	48.1%	108.9%	79.7%	106.0%	82.9%
**M**	**30.7%**	**22.9%**	5.1%	25.0%	11.8%	28.3%	12.0%	14.3%	71.6%	25.5%	53.1%	21.2%
**MP**	173.2%	**28.3%**	173.2%	NA	173.2%	NA	173.2%	141.4%	173.2%	NA	173.2%	NA
**P**	123.2%	126.0%	164.8%	139.3%	32.1%	137.4%	103.7%	141.4%	157.6%	107.0%	157.6%	105.1%
**S1**	99.8%	113.1%	173.2%	141.4%	152.4%	141.4%	173.2%	NA	NA	141.4%	NA	141.4%
**TB**	69.3%	**28.3%**	NA	141.4%	NA	NA	NA	NA	173.2%	NA	173.2%	NA
**W1**	NA	**28.3%**	NA	NA	NA	141.4%	NA	NA	NA	NA	NA	NA
**X2**	106.3%	43.7%	15.3%	83.6%	90.3%	26.5%	79.4%	38.2%	173.2%	31.3%	173.2%	39.2%
**X3**	102.9%	141.4%	86.7%	141.4%	134.7%	141.4%	113.2%	141.4%	89.0%	141.4%	91.1%	141.4%
**Y**	62.9%	74.5%	83.3%	61.7%	20.1%	84.9%	52.3%	4.4%	88.0%	108.5%	70.9%	112.9%

1. Padisak, J.; Crossetti, L.O.; Naselli-Flores, L. Use and misuse in the application of the

phytoplankton functional classification: a critical review with updates. *Hydrobiologia* **2009**, *621*, 1-19.

2. Reynolds, C.S.; Huszar, V.; Kruk, C.; Naselli-Flores, L.; Melo, S. Towards a functional classification of the freshwater phytoplankton. *J Plankton Res* **2002**, *24*, 417-428.
